# The Treatment of Pyorrhea Alveolaris, with Special Reference to Emetine

**Published:** 1915-04

**Authors:** Robin Adair

**Affiliations:** 319 Grant Building, Atlanta, Ga.


### Journal-Record of Medicine

Successor to Atlanta Medical and Surgical Journal, Established 1855
and Southern Medical Record, Established 1870

**OWNED BY THE ATLANTA MEDICAL JOURNAL COMPANY**

PUBLISHED MONTHLY

*Official Organ Fulton County Medical Society, State Examining Board, Presbyterian Hospital, Atlanta, Birmingham and Atlantic Railroad Surgeons' Association, Chattahoochee Valley Medical and Surgical Association, Etc.*

EDGAR BALLENGER, M. D., Editor
BERNARD WOLFF, M. D., Supervising Editor
A. W. STIRLING, M. D., C. M., D. P. H.; J. S. HURT, B. Ph.. M.D.
GEO. M. NILES, M. D., W. J. LOVE, M. D., (Ala.); Associate Editors
R. R. DALY, M. D., Associate Editor
E. W. ALLEN, Business Manager

**COLLABORATORS**

W. F. WESTMORELAND, M. D., General Surgery
F. W. McRAE, M. D., Abdominal Surgery
H. F. HARRIS, M. D., Pathology and Bacteriology
E. B. BLOCK, M. D., Diseases of the Nervous System
MICHAEL HOKE, M. D., Orthopedic Surgery
CYRUS W. STRICKLER, M. D., Legal Medicine and Medical Legislation
E. C. DAVIS, A. B., M. D., Obstetrics
E. G. JONES, A. B., M. D., Gynecology
R. T. DORSEY, Jr., B. S., M. D., Medicine
L. M. GAINES, A. B., M. D., Internal Medicine
GEO. C. MIZELL, M. D., Diseases of the Stomach and Intestines
L. B. CLARKE, M. D., Pediatrics
EDGAR PAULLIN, M. D., Opsonic Medicine
THEODORE TOEPEL, M. D., Mechano Therapy
A. W. STIRLING, M. D., Etc., Diseases of the Eye, Ear, Nose and Throat
BERNARD WOLFF, M. D., Diseases of the Skin
E. G. BALLENGER, M. D., Diseases of the Genito-Urinary Organs

VOL. LXII. ATLANTA, GA., APRIL, 1915. NO. 1

### THE TREATMENT OF PYORRHEA ALVEOLARIS, WITH SPECIAL REFERENCE TO EMETINE.

ROBIN ADAIR, M. D., D. D. S.

At birth the mucous membrane in the mouth affords the same protection as in the other parts of the body. As each tooth is erupted, a hole is punctured through this protective covering, in all, thirty-two holes. Although the tooth fills this opening, the margin is always open, as long as a tooth is standing in the jaw. In other words, the mucus membrane is not attached to the tooth, and leaves, as it were a crack into which the flora and parasites of the mouth are free to enter. Where the mouth is in a healthy condition, this crevice is very slight, but the dirtier the mouth, the wider it becomes, with an increasing load of infection. Thus it is easy to see why extraction of a Pyorrhea tooth seems so quickly to cure. The irritant being removed, further protection takes place on account of the mucus membrane closing over the opening where the tooth stood.

Black has said that the human teeth bear something like 1700 pounds of pressure each day. If we will liken a tooth in its socket to a piston working under a heavy load, we will see how easy it is for bacteria and its products to be forced into the broken blood vessels found here and the cancellous structure of the jaw bone. Estimating the infected surface of a Pyorrhea case, we are surprised to find its extent from three to seven square inches. In addition to this, if we will compute the quantity of pus and other poisonous products excreted and swallowed daily by the average case of Pyorrhoea, something like a half teaspoonfull will be a safe average. The wonder of it all is that nature can provide immunity against such odds. You can safely tell your patients, however, that just as sure as death and taxes, sooner or later, they are to suffer from this gradual pumping into the system and swallowing of these poisons. Goadby, one of our best authorities on Mycrology of the mouth, has said, "small, but considerable dosage with bacteria and products from a local focus tends to gradually break down immunity."

The micro-organisms in Pyorrhea are well protected, due to the dense membrane which they inhabit, to the large amount of granulating tissue present, calculi, and the further fact that the parts here are so poorly supplied with blood vessels. The blood contains special ferments which are destructive to bacteria, but, because of the above mentioned protection, they cannot get into the pocket or fort held by the bacteria. If this were not the case, we would probably not have any

Pyorrhea infection, for it is rarely a case of primary blood infection occurs. For this reason, we must see that the only logical treatment of this disease must be to either extract the tooth, or to surgically remove this culture bed of infection.

Nearly all mouths contain the Pneumoccus, whether this is the true Pneumoccus is a debatable question. We do know that some of the worst infections and complications from Pyorrhea are due to its presence. Post-operative Pneumonia is due in many cases to Pyorrhea mouths having a super-quantity of these micro-organisms.

The Treponema mucosum is a new species of Spirochæta reported by Dr. Noguchi. This organism presents an appearance much like that of syphilis. Its action is Pyogenic and it will not grow in healthy tissue. The peculiar odor of Pyorrhea is produced by this organism.

Dr. E. C. Rosenow has opened up a new field for those who are interested in the bacteriology of the mouth. His discovery was the transmutation by animal passage of the streptococcus viridans into pneumoccus. He explains that the organism in rheumatism locates in joints and endocardium because of the low degree of oxygen-pressure in that these structures are very avascular in that here we meet the end of the capillary supply. The same infection by the process of convertion may show streptococcus viridans in endocarditis and hemolytic streptoccus in the joints. However, the point in his investigation of most importance to the dentist is his statement as to the extent of infection and the place where these affinities occur. "It is safe to say that the focus is most commonly found in the oral cavity and here in order in frequency is probably the tonsil, pyorrhea and blind abscesses about the teeth."—Journal Lancet.

DISEASES DUE TO PYORRHEA.

Nor will space here allow the insertions of full case records from the specialists, but the dental and medical literature is full of them. That the beginner in this work may know what great benefit may be given humanity, mention is made of a few results, selected at random from authoritative reported cases.

Cases of arthritis deformens, due to Pyorrhea, have been greatly benefitted by dental attention. Ulcer of the stomach, caused by Pyorrhea, cured by dental treatment. Many cases of chronic dyspepsia have yielded promptly. Patients who had albumen and casts inside of two weeks have been returned to normal conditions.

Dr. Upson has reported that Pyorrhea has caused many cases of insanity and that a large number of such cases are restored by curing the Pyorrhea.

From these examples, we would not have our medical friends think that we are claiming the whole of pathology is caused by Pyorrhea, or that its treatment is a "cure all," but we will admit that it seems each month to be tending that way, due to the recent startling discoveries, all of which goes to show the intimate relationship which could exist between the two professions.

Dyspepsia, flatulence and gastric ulcer, together with all their varied symptoms, are often caused by the large numbers of bacteria and their products which are constantly swallowed.

The tonsils, bronchi and even the lining membrane of the arteries and heart may become infected from diseased gums. Many cases of eye and ear complications are relieved by curing the case of Pyorrhea.

Patients will often want to know how it is that certain of their friends have had Pyorrhea for twenty-five years, and seem to get along without any inconvenience. In answer, we may assure them that the immunity is more in appearance than real, and that at any time this immunity may suddenly give way, and any of the mentioned complications arise too late for a cure.

Dr. Julian Zilz, of Vienna, reports some interesting statistics about the frequency of the association of diabetes with Pyorrhea Alveolaris. Seventy-one of the cases examined had Pyorrhea Alveolaris. Nearly half of these have a history of gum trouble before the diabetes. Only in a small number did the Pyorrhea follow the incipiency of the diabetes. In all these cases, the saliva showed acid reaction. This report shows the necessity of making the sugar test with a specimen of the patient's urine.

Many investigators has attributed auto-intoxication as the cause of many cases of Pyorrhea. As above mentioned, intestinal complications should always be suspected and the proper attention given thereto.

The large amount of filth, germs and parasites in a Pyorrhea mouth which is constantly swallowed or mixed with food must in time affect the stomach or intestines. It is not probable that the secretions of the stomach can at all times handle this material from the mouth. When it is passed into the intestines some of this toxic matter is absorbed, their structures may become diseased, or the blood become saturated with some form of this toxic matter.

On the other hand, there is as much argument to support those who start with a sluggish liver, inactive kidneys, constipation, germ-infected fecal matter, inadequate elimination with its resultant auto-intoxication thrown into the blood stream to afterwards break out at some location, as for instance, the gums around the teeth. But the question comes, would it do so unless the gums were already diseased? No matter which way we believe, the condition is serious enough for consultation with a physician who should recognize the gravity of the situation and co-operate with the dentist by prompt and energetic treatment, for there is nothing we can do to hasten the cure of a case of Pyorrhea under treatment as much as the re-establishment of the proper elimination process of the intestines. Bear in mind that auto-intoxication generally occurs in those who indulge in excesses of food and drink. Reduce the foods containing animal proteids. Meat must be forbidden as a food. Fresh butter-milk or some of the artificial varieties must be taken in large quantity to reduce the fermentative process in the intestine by its anti-fermentative property. Insist that the patient drink a large quantity of water, preferably lithia, each day. The commercial houses by their attractive literature, suggest many wonder-working preparations, but just plain old epsom salts will do the work. Begin by prescribing one tablespoonful in water every other morning for four mornings, then reduce to one teaspoonfull each morning for one week.

Dr. B. Craig, chief of clinic Neurological Institute of New York, in a paper before the American Medical Association, made the following statement:

"For a considerable period it has been observed that a bad condition of the teeth may be responsible for adjacent disorders, such as otalgia and tic douloureux, but the observation that stubborn neuritis of the sciatic or other nerve trunk or of the brachial plexus sometimes disappears after cleaning out the alveolar disease, is of comparatively recent date. The continual swallowing and absorption of pus is undoubtedly the cause of disorders of digestion, headache and finally an anemic condition almost cachectic. This depleted, exhausted state may often be associated with a melancholic state. It seems a far cry from mouth infection to mental disease, but when one witnesses profound depression clear up following the drainage of several alveolar pus-pockets, one is persuaded that the chronic intoxication, the result of absorption from the pent-up infectious process, was an etiologic factor."

### THE EMETINE TREATMENT.

Since scientific investigations have been in vogue with the dental profession, since we have known anything about bacteriology, every once in a while some investigator claims that certain germs or parasites are responsible for diseased conditions of the mouth. However, none of these have had the success of the recent investigators, Barrett, Smith, Bass and Johns. Like a bolt from a clear sky, before the dental profession could make investigations properly, the laity papers throughout the land, and the Medical Journals proclaimed that at last the absolute cause of Pyorrhea had been found and its sure cure was at hand. Inside of three months, the pharmaceutical houses all over our land got out nicely printed literature containing the reports of these gentlemen on the Endameba Buccalis, claiming that they had the particular form of ipecac which was to be our salvation in its cure. As soon as some of us could catch up with this rapid development, and had begun to catch our breath for investigation, we found that every dentist in the land was spending a larger amount of money for Emetine than they had ever spent to cure Pyorrhea before, and on investigation, few, if any, saw any improvement in the cases treated. Still, they seemed stampeded by the idea, not one seemed to be willing to advance other views, or to raise a voice about the failures he had had.

In one case, a physician read a paper before a Dental Society and one dentist attempted to question the miraculous cures reported, but he was wafted aside with the statement that he was yet in the Amoeba state and had not developed the technique for curing Pyorrhea. I would not for a moment decry any new movement or discovery, and no one is working harder on experiments and practical cases to keep up with every new development than myself, but I do deem it unfortunate indeed that this new development was thrust upon the profession just at the time when we had begun to work and master the surgical technique necessary to save these teeth, and now comes, these gentlemen, deriding such means as having failed. Surely it is to be hoped that this attempt to throw aside our successful technique will be short lived.

It is rather annoying that now, only about three months after the first announcement of Ipecac as a cure for Pyorrhea, if a dentist attempts to treat a patient without using it, his attention is immediately called to the omission by the patient.

Of course, in a report of this kind, this matter must be brought down to its latest form. First, we must remember that the bacteria of the mouth are flora, of vegetable origin, like wheat or corn. These are microscopical in form. Many years ago, investigators found in the secretions of very dirty mouths, unicellular bodies, larger bodies, which were not of the flora form, but parasitic, these were given the name of Endameba Buccalis. The gentlemen above referred to, about the same time in Philadelphia and New Orleans, found more of these in Pyorrhea mouths than in normal mouths, this lead them to the conclusion that this was the cause of Pyorrhea. Of course, these Amoeba can be seen moving about in the microscopic field, also a large amount of bacteria, then it is a question what the amoeba are there for, whether they intend to destroy the alveolus or gum structure, or have only crawled into these pockets for a meal of bacteria, for this reason it is a question whether or not the amoeba is pathogenic to human tissue. It is my opinion that the parasytes are normal residents in even the mouths of children, and the greater the bacterial coat, the greater the filth in the mouth, the easier they can propagate. In their enthusiasm due to their findings, the investigators fail to produce sufficient proof of the amoeba as a causative factor. For instance, I might say that blue eyes cause Pyorrhea. (I might be of sufficient importance to have it heralded all over the country), saying that fifty per cent of the people affected with Pyorrhea have blue eyes, therefore, blue eyes are the cause, and, further, prove the statement by saying that many people who do not have blue eyes do not have Pyorrhea. So it is with the Endamoeba Buccalis, they are found in Pyorrhea mouths, therefore they cause this disease. I flesh of this carcass might be present animal parasytes, then I might be passing through the fields and find a carcass, in the would go on a little farther and find another carcass and in the flesh of this one also might be present more of the same parasytes, also I could remember that years ago I had seen the same thing, then I might immediately draw the conclusion that these parasytes were the cause of the death of the animals, never going back to investigate if some other agent had caused the break in the continuity of the life of the animals. If we could keep the Pyorrhea mouth free from germs, we would probably not find the amoeba present in as large quantities.

It seems to be necessary to recall to the minds of our medical friends that the dental profession has for years been curing Pyorrhea and that this has been accomplished by performing the proper surgical work and after proper similation of the structures and only after these procedures, have we cured

Pyorrhea and then, without regard to Amoeba. The method used was to remove this coat of bacteria and then the tissues given proper stimulation, the patient has been cured of Pyorrhea.

Can anyone look at the dead peridental membrane on an extracted Pyorrhea tooth, can anyone look at the skull of a dead person who had Pyorrhea and see the bone destruction there, can anyone after seeing these sights, say that he believes the application of any drug could remove the cause of Pyorrhea and cure it? Thus we do not underestimate the importance of Amoeba in Pyorrhea cases, but we must discredit the reports of the marvelous cures from the use of Ipecac only.

Does it have any bearing on the facts in the case to say that a man having Amoebic Colitis was given one hundred sixty grains of Ipecac without any sign of improvement of the diseased mouth? Does it have any bearing on the case to say that within one month after this, when his mouth had been cleaned up, the disease was eradicated?

Does it have any bearing on the case to state that when I have a splinter in my finger it suppurates, then I might by the injection of Emetine arrest the suppuration for a few hours, if I should still leave the splinter in the finger, a relapse would occur? This is the same relapse that is so often spoken of by the investigators who treat Pyorrhea by Emetine. The cause is still there in both cases and must be removed before a permanent cure can be effected.

I have used Emetine, am using it, and shall continue to use it, but it is on thorough surgical work that I depend for my cures. In reading the various advertisements of the pharmaceutical houses not only of Emetine, but for other drugs also, I have been struck with the emphasis which has been put on the statement that they are absolute cures without any surgical help. It remainds me of once when I looked through an automobile magazine at the various advertisements in regard to appliances for saving gasoline, every one claimed from forty to fifty per cent saving. I figured up that by using every one advertised I would run my car without water, oil or gasoline.

However, I still find it necessary to have plenty of all of these before starting out on a pleasure ride. Thus it is just as necessary for us to go back to thorough instrumentation as it is to have oil, gasoline and water to run an automobile, that is until some other agent now unknown, is discovered for the treatment.

Read the recent papers by Dr. Bass and compare his claims and findings with a paper read before the New York Pathological Society, 1907, by Dr. L. T. LeWald, in which he showed amobae could be demonstrated in the mouth almost constantly, no matter how much care was taken of the teeth.

The Bureau of Laboratories of the New York Health Department will next month publish their report on "Amoebas in the Mouths of School Children." If Dr. Bass' claims are true, then all school children have Pyorrhea. Such a claim is absurd on the face of it.

Does it have any effect on the status of Ipecac Treatment when we read that Drs. Merritt, Hartzell, Talbot, West, McCall and other distinguished investigators in the field of Pyorrhea could not verify the results claimed for this treatment?

A distinguished medical investigator, Rosenberger of Jefferson Medical College, chair of bacteriology, writes me as follows: "The ameba buccalis plays very little, if any part in the etiology of Pyorrhea. * * * My own results with Emetine have been very poor."

The distinguished Rosenow, of Chicago, writes me under recent date that "the work of Bass and Barrett in the use of Emetine in the treatment of Pyorrhea is very interesting, but I fear that they have not sufficiently emphasized the need of proper dentistry in the way of removal of tartar, deposits, etc., for the permanent relief."

The greatest mistake made by Dr. Bass is that he declares every individual has Pyorrhea, just because he believes the Ameba is the cause of Pyorrhea, and he finds that in all mouths. Dirty, filthy mouths have the largest number of these parasites, and it is a strange fact that just such a mouth may be free from Pyorrhea. On the other hand, Pyorrhea may be present in a well kept mouth. As Pyorrhea Alveolaris is an affection of the peridental membrane of the tooth with the formation of tartar and the molecular death of the alveolar process as its last stage. The most severe cases may not have any pus pockets.

Many physicians have of late been giving their patients Emetine by the hyperdermic method without a proper diagnosis for Pyorrhea. Some have come into my office with both arms so sore they could not do their ordinary duties. Some of these had no Pyorrhea. I have seen ulceration of the mucus membrane of the mouth due to the physician prescribing Ipecac as a mouth wash. The writer urges physicians to examine and diagnose Pyorrhae, but believes the co-operation of a dentist in the treatment will result in more good for the patient.

It is our custom to make a physical examination, the record of which is kept in a special book. A comparatory table of the temperature, blood-pressure and urinalysis, taken before and after treatment, proves the ultimate association with other diseases, as described in the first part of this paper.

We generally find that the patient has fautly elimination or indicanuria, and does not eat the proper food. If we find any systematic disease, we refer patient to a competent physician to co-operate with us in restoring the patient to health.

The proper surgical work for Pyorrhea is most exacting, and requires much skill. Regardless of the claims of Barrett, Bass and others, the most successful cure of Pyorrhea is dependent entirely upon the thoroughness with which this work is done.

The field is sterilized, the roots of the teeth planed, and the pockets curetted. We always endeavor to leave in the pockts a fresh blood-clot which we protect until it becomes new structure.

When the teeth become comparatively firm, and the mouth is in a sanitary condition, the patient is taught the proper care of the teeth. If this instruction is carried out by the patient, he should be able to keep his mouth in a sanitary condition.

Those dentists who are using the above method have been successful in their treatment of Pyorrhea.

319 Grant Building, Atlanta, Ga.

